# Socioeconomic Status and Risk-Taking Behavior Among Chinese Adolescents: The Mediating Role of Psychological Capital and Self-Control

**DOI:** 10.3389/fpsyg.2021.760968

**Published:** 2021-12-06

**Authors:** Xiaoshan Jia, Haidong Zhu, Guiqin Sun, Huanlei Meng, Yuqian Zhao

**Affiliations:** ^1^Normal College, Shihezi University, Shihezi, China; ^2^Psychological Application Research Center, Shihezi University, Shihezi, China

**Keywords:** socioeconomic status, risk-taking behavior, psychological capital, self-control, adolescents

## Abstract

Risk-taking behavior is particularly widespread during adolescence, and negatively impacts the healthy growth and social adaptation of adolescents. Utilizing problem-behavior theory (PBT) and the family stress model (FSM), the current study examined the relationship between socioeconomic status (SES) and adolescents’ risk-taking behavior, as well as the mediating role of psychological capital and self-control. A total of 1,156 Chinese adolescent students (M_age_ = 15.51, 48% boys) completed a series of questionnaires anonymously. The results showed that: (1) Socioeconomic status was negatively correlated with adolescents’ risk-taking behavior; (2) Both psychological capital and self-control mediated the relationship between SES and adolescents’ risk-taking behavior independently; and (3) Psychological capital and self-control also mediated the relationship between SES and the risk-taking behavior of adolescents sequentially. This study reveals the internal mechanism of risk-taking behavior during adolescence and provides theoretical support and empirical evidence for preventing and reducing such behavior in this age group.

## Introduction

Risk-taking behavior refers to action taken by an individual to obtain beneficial results when he/she can perceive the negative consequences of an intentional behavior (Ben-Zur and Zeidner, 2009). This form of behavior can be divided into positive and negative types. The former refers to behaviors that are beneficial to physical and mental health and are acceptable to society, such as mountaineering, skiing, participating in speech contests, and so on. The latter refers to intentional actions that endanger the physical and mental health of oneself and others. These are also known as maladaptive or problem behaviors, such as smoking, drinking, violent attacks, and other crimes (Özmen and Sümer, 2011; Duell and Steinberg, 2020). Adolescence is typically characterized by a high incidence of risk-taking behavior. The occurrence of negative risk-taking behavior (hereinafter referred to as risk-taking behavior) may severely hinder the healthy growth of adolescents and their subsequent social adaptation (Gardner and Steinberg, 2005; Smith et al., 2014). Recent studies have found that health-risk behaviors such as smoking, drinking, violence, and violations of regulations have gradually increased among Chinese adolescents, who now account for a growing proportion of criminal behavior (Ren and Lv, 2016; Yuan and Wen, 2019). The extent of the issue demands that both the risk and protective factors involved in such behavior are investigated in order to develop approaches that can better support the physical and mental health of adolescents.

The important factors involved in risk-taking behavior are environmental, psychological, and biological. Environmental factors include the family, peers, school, and the neighborhood (Steinberg, 2016). Personal inner factors include individual beliefs, values, motivations, and goals (Rolison and Scherman, 2003; Mcghee et al., 2012). Biological factors mainly refer to the effects of changes in brain structure and function during adolescence (Steinberg, 2008). According to problem-behavior theory (PBT), an individual’s social behavioral tendencies are formed by the interaction of the environmental, individual inner and behavioral factors, and any change in these factors will alter behavior (Jessor, 1987). Environmental and psychological factors may be relatively stable, but some phenomena peculiar to adolescence, the second peak of physical and mental development, may exacerbate preexisting risk-taking tendencies, potentially leading to more serious and irreversible consequences. It is thus of great significance to explore how the environment, inner factors and the specific physiological changes of adolescence interact with each other and how these factors related to the development of risk-taking behavior during adolescence.

### Socioeconomic Status and Risk-Taking Behavior

As one of the main family environmental variables, socioeconomic status (SES) plays an important role in the development of individuals. SES can be defined as the relative wealth, power, and social status possessed by individuals, families, or collectives (Mueller and Parcel, 1981). It can be divided into two types: objective SES and subjective SES. For teenagers, the objective SES includes parental education, occupation status and family income; the subjective SES is the subjective perception of the individual’s own SES (Kraus et al., 2011a). Although the two types of SES have a moderate correlation, many studies have shown that there are differences in the relevant research results of objective and subjective standards of SES (Adler et al., 2000; Chen et al., 2014). Therefore, some researchers suggest that the two types should be included to reflecting adolescents’ SES more comprehensively and effectively (Kraus et al., 2011b; Tan and Kraus, 2015).

The family stress model (FSM) holds that parents in families with lower SES experience greater economic and psychological pressures, and are less able to devote excess energy to educating their offspring, which leads to more psychological and behavioral problems in children (Conger et al., 2010). This may be because the adverse effects of low SES environments are widespread. Generally, increased economic pressures on parents impact the family environment by exerting greater psychological pressure on its members. These are manifested in negative parenting styles, difficulties in parent-child relationships, and problems communicating with peers. Moreover, these factors will exacerbate the consequences of adverse conditions on the development of adolescents, because families of lower SES tend to cluster in communities and schools with similar conditions (Conger and Elder, 1996; Masarik and Conger, 2017). Research suggests that adolescents from families with lower SES have a greater propensity for substance abuse (Bersamin et al., 2017; Lee et al., 2017), overeating (Lee et al., 2013), unsafe sex (Vukovic and Bjegovic, 2007), violating social rules (Piotrowska et al., 2015) and other risk-taking behaviors. Therefore, lower SES may be a risk factor in adolescents’ risk-taking behavior. To date, studies on the relationship between SES and risk-taking behavior have focused on Europe, the Americas, and other extremely deprived areas elsewhere, with the issue relatively unexplored in the Chinese context. However, several previous studies have concluded that SES is negatively correlated with Chinese adolescents’ health-risk and problem behaviors (Cui and Lou, 2019; Liu et al., 2020), while other research detected no significant correlation (Wang et al., 2018). These mixed results indicate the need to clarify the nature of the relationship between SES and Chinese adolescents’ risk-taking behavior in order to determine the presence of cross-cultural consistency in the relationship between the two. Therefore, the first hypothesis of this study is that SES is negatively associated with adolescents’ risk-taking behavior (H1).

### Socioeconomic Status, Psychological Capital, and Risk-Taking Behavior

Personal inner factors are more important than environmental factors to adolescents’ risk-taking behavior. Scholars have demonstrated that inner factors including self-esteem, self-efficacy (Rosenthal et al., 2010), resilience (Titterton and Smart, 2010), the Big Five personality (Hong and Paunonen, 2009), self-regulation and sensation seeking are all closely related to risk-taking behaviors in adolescence (Steinberg et al., 2017). Moreover, positive psychology’s focus on the healthy growth and self-fulfillment of individuals has led researchers to investigate the role of individual psychosocial capacity. As a comprehensive embodiment of individual psychosocial capacity, psychological capital is a positive psychological state shown by individuals in the process of growth and development, which is characterized by stability and plasticity and includes the four core components of self-efficacy, resilience, hope, and optimism (Luthans et al., 2007). A high level of psychological capital counteracts depression, anxiety, and other negative emotional distress (Xiong et al., 2020), and reduces the possibility of substance abuse, unsafe sexual activity, assault, crime, and other problem behaviors (Ludwig and Pittman, 1999; Malti and Noam, 2009; Rew et al., 2017). It was also found to enhance social adaptability and protect or improve adolescents’ physical and mental health (Finch et al., 2020).

The accrual of adolescents’ psychological capital is closely associated with the family’s SES. Long-term low SES weakens the psychological capital of adolescents and may indirectly account for subsequent psychological and behavioral problems (Goosby, 2007; Schelleman-Offermans and Massar, 2020). While the influence of SES on the psychological and behavioral changes of adolescence is often mediated by individual internal factors (Bradley and Corwyn, 2002), most previous studies have only considered the mediating effects of certain elements of psychological capital on the relationship between SES and risk-taking behavior (Lansford et al., 2006; Layte and Whelan, 2009). However, the effect of SES on positive individual traits is undoubtedly multi-faceted; a comprehensive investigation of psychological capital as a mediator will enable to be better understand the relationship between SES and psychological and behavioral changes of adolescence. Therefore, this study speculates that psychological capital plays a mediating role in the relationship between SES and adolescents’ risk-taking behavior (H2).

### Socioeconomic Status, Self-Control, and Risk-Taking Behavior

Self-control is an important personality trait that affects adolescents’ risk-taking behavior. It is defined as an individual’s efforts to control and adjust their behavioral tendencies to meet social expectations or self-standards (Tangney et al., 2004). In this regard, it is useful to consider Gottfredson and Hirschi’s general theory of crime, which suggests that individuals with poor self-control will take more risks and engage in socially deviant behavior, including criminal acts (Gottfredson and Hirschi, 1990; Shoenberger and Rocheleau, 2017). However, for adolescents, the neurobiological changes that occur in adolescence must also be acknowledged. Researchers believe that youth risk-taking behavior is a product of the interaction between the brain’s “socioemotional” and “cognitive control” systems. Whereas the socioemotional system develops rapidly during puberty, increasing the drive toward stimulation and reward-seeking, the cognitive control system develops at a slower rate, meaning that self-regulation and impulse control are relatively poor. This mismatch increases the likelihood of risk-taking during adolescence (Steinberg, 2008, 2010). Therefore, the development of self-control—which is closely related to higher cognitive processes such as planning, regulating attention, predicting outcomes, and restraining impulses—may be importantly related to adolescents’ participation in risk-taking behaviors. Subsequent studies have also confirmed that adolescents with low self-control are less able to self-regulate and suppress impulsive risk-taking tendencies, making them more likely to engage in behaviors such as smoking, drinking and fighting (Cheung, 2014; Margot et al., 2017; Tian et al., 2018).

Moreover, SES has been closely tied to the development of adolescents’ ability to exercise self-control (Farley and Kim-Spoon, 2017). Analyzing the causes of juvenile delinquency in China, Zheng and Luo (2009) found that criminal behaviors occurred primarily in youth groups with low self-control, which was significantly related to the SES of the family. Recent studies on brain mechanisms have also implicated cognitive control in demonstrating that SES has a significant indirect impact on changes in adolescents’ risk-taking behavior. Adolescents from families with lower SES exhibit lower levels of cognitive control, increasing their tendency to take risks (Brieant et al., 2020). It is therefore likely that poor SES exacerbates the negative effects of low self-control on risk-taking behavior during adolescence. On this basis, the present study speculates that self-control mediates the relationship between SES and adolescents’ risk-taking behavior (H3).

### Socioeconomic Status, Psychological Capital, Self-Control, and Risk-Taking Behavior

However, the connection between psychological capital and self-control requires further investigation. Does it associate with adolescents’ risk-taking behavior independently or continuously? According to Luthans et al. (2015), the improvement of self-control is likely to result from active mobilization of the internal resources possessed by individuals. Psychological capital contains four kinds of positive psychological resources, which play an important role in the maintenance of self-control. Previous studies focused on the relationship between the components of psychological capital and self-control. Self-efficacy has a positive and direct relationship with self-control, and adolescents could adjust their efforts and time allocation according to their expectation of behavioral results (Mabekoje, 2010). The implementation of resilience builder program for adolescents improved their ability to regulate attention, emotion and behavior (Johnson, 2012). Positive affect such as optimism and hope could also enhance adolescents’ self-control, even in the context of ego depletion (Tice et al., 2007). The above results indicate that each component of psychological capital is positive associated with self-control, but the relationship between psychological capital and self-control is rarely studied as a domain-general positive psychological resource. Based on previous researches, we believe that psychological capital can support individuals’ self-control and improve their performance on important tasks, while the capacity to apply self-control is limited by the psychological capital available to each individual (Baumeister et al., 2007). Therefore, this research speculates that psychological capital and self-control mediated the relationship between SES and the risk-taking behavior of adolescents sequentially (H4).

**FIGURE 1:**
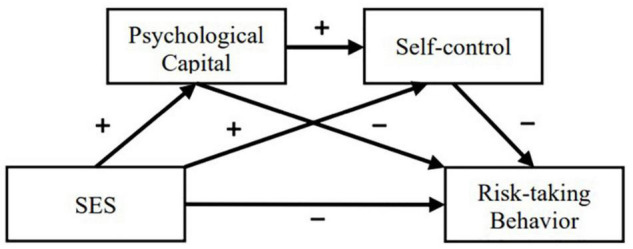
Conceptual model.

In summary, the current study systematically examines the mediating role of psychological capital and self-control in the relationship between SES and adolescents’ risk-taking behavior, including their independent and serial mediating effects. Ultimately, this will explain the mechanism of the relationship between SES and adolescents’ risk-taking behavior. At the same time, the results of this study will provide theoretical support and empirical evidence for preventing and intervening in adolescents’ risk-taking behavior. The concrete conceptual model is shown in Figure 1.

## Materials and Methods

### Participants and Procedure

This study was approved by the Ethics Committee of the author’s research institution, and informed consent was provided by all stakeholders, including teachers and other school staff, parents, and students. A group of adolescents aged between 11 and 19 from four schools in the northwest of China was selected to participate. Pre-trained psychology graduate students administered the questionnaires and answered students’ questions about the survey. Student participation was entirely voluntary and all respondents completed the questionnaires anonymously during self-study classes. A total of 1,280 questionnaires were distributed, of which 1,156 valid questionnaires were returned. The mean age of these participants was 15.51 years (*SD* = 2.27), with the sample comprising 554 boys and 602 girls.

### Measures

#### Socioeconomic Status

In line with previous research (Tan and Kraus, 2015; Li X. X. et al., 2019), the participants’ family SES was taken to be that of their SES. The status was measured *via* several objective and subjective indicators, which were converted into standardized scores and added together, reflecting the participants’ SES more effectively.

To calculate the participants’ objective SES, the parental education, occupation, and family property resources were considered, drawing on the methods presented in Zhou and Guo (2013) and Chen et al. (2014). Parental education was measured on a six-point scale, with the six categories comprised of elementary school or below, junior high school (including non-graduates), high school or technical school (including non-graduates), college, university graduate, and post-graduate level. Parental occupation was determined on the basis of the ten major social classes proposed in Lu (2002) and measured on a corresponding 10-point scale. To measure family resources, the participants were asked to report on the availability of 14 key objects (such as their own room, desk, and computer) in their households, with responses scored between 0 and 14 points. Only one parent with the higher educational and occupational level was considered when calculating the objective SES. The three indicators above were then converted into standardized scores and principal component analysis was performed to obtain the main factor coefficient, with an eigenvalue of >1. After calculating the factor loading for each of the three indicators, the overall SES score for each participant was calculated according to the formula [Objective SES = (β_1_ × Z_Education level_) + (β_2_ × Z_Occupation_) + (β_3_ × Z_Family property resources_)/εf]. Among them, 0.81, 0.83, and 0.71 are the factor loadings of the three indicators, respectively, and 1.85 is the eigenvalue of the main factor, with higher scores corresponding to higher SES. In this study, the objective SES scores ranged between −2.70 and 2.71.

Subjective measures of status were determined using the MacArthur Scale of Subjective SES (Adler et al., 2000; Shang et al., 2016). Participants were asked to evaluate their family’s SES based on parental education, occupation, and family’s financial situation by marking the appropriate rung on a 10-level ladder, with the lowest status at the foot and the highest at the top of the ladder.

#### Psychological Capital

This variable was measured using the Positive Psychological Capital Questionnaire (Zhang et al., 2010), which is widely used to measure the psychological capital level of Chinese participants. This questionnaire includes 26 items on the four dimensions of self-efficacy, resilience, hope, and optimism. One example was the statement, “Many people appreciate my talents.” Each item was scored on a 7-point scale ranging from 1 “completely inconsistent” to 7 “completely consistent”; higher scores indicate more advanced levels of psychological capital. A Cronbach’s Alpha of α = 0.84 was recorded for this instrument.

#### Self-Control

The Chinese version (Tan and Guo, 2008a,b) of the Self-Control Scale (SCS; Tangney et al., 2004) was used to measure the participants’ levels of self-control. It includes five dimensions (resistance to temptation, healthy habits, task performance, impulse control, and moderate entertainment) and consists of 19 items overall, one item of which is “I can resist temptation well.” Each item was rated on a 5-point scale ranging from 1 “totally disagree” to 5 “totally agree.” Participants who scored higher on this scale demonstrated a greater capacity to exercise self-control and the scale’s internal consistency was strong (α = 0.86).

#### Adolescents’ Risk-Taking Behavior

The original version of the Adolescent Risk-Taking Questionnaire (Gullone et al., 2000) was later translated and revised by Zhang et al. (2011) for use with Chinese adolescents. The scale consists of 17 items and includes four dimensions: thrill-seeking, rebellious risk, reckless risk, and anti-social risk. In line with previous research and the purpose of this study, the last three dimensions were used to measure negative risk-taking behavior (Liu et al., 2019), with items such as “Ride a bike after drinking.” Responses to the questionnaire were graded on a 5-point scale, ranging from 0 (“never”) to 4 (“always”). Higher scores indicated greater involvement in negative risk-taking behaviors (α = 0.86).

#### Data Analysis

The common method bias test, descriptive statistics, and correlation analysis of the study data were conducted using SPSS 22.0. The conceptual model was tested using Model 6 of the SPSS PROCESS macro. Finally, according to the Bootstrap test procedure, the data was resampled 5000 times, enabling the size of each mediating effect to be calculated at a 95% confidence interval.

## Results

### Test for Common Method Bias

Owing to the study’s reliance on self-report, the possibility of common method bias was investigated. Harman’s single factor test was used to determine the existence of 16 factors with eigenvalues greater than 1; the variance explained by the first factor accounted for 17.61% of the total variance, well below the specified standard of 40%, indicating that common method bias was not an issue in the study.

### Descriptive Statistics and Correlation Analysis

Table 1 presents the descriptive statistics and results of the correlation analysis. There were significant positive correlations between SES, psychological capital, and self-control, and significant negative correlations between SES, psychological capital, self-control, and risk-taking behavior. Because gender and age were significantly related to the main research variables, they were controlled for in the follow-up analysis.

**TABLE 1:** Descriptive statistics and correlations between variables.

	*M*	*SD*	1	2	3	4	5	6
1. Gender	0.52	0.50	−					
2. Age	15.51	2.27	−0.05	−				
3. SES	0.02	1.58	0.08**	−0.045	−			
4. Psychological capital	118.27	10.49	−0.04	−0.048	0.30**	−		
5. Self-control	65.86	9.13	−0.02	−0.29**	0.25**	0.43**	−	
6. Risk-taking behavior	7.02	2.48	−0.14**	0.25**	−0.19**	−0.27**	−0.39**	−

Gender: 0 = male, 1 = female; **p < 0.01.

### Test for Multiple Mediation Model

**FIGURE 2:**
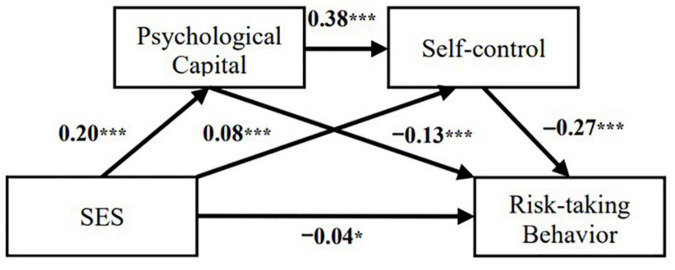
Multiple mediation effect model. *p < 0.05 and ***p < 0.001.

The mediating role of psychological capital and self-control in the relationship between SES and risk-taking behaviors were measured using Model 6 in the SPSS PROCESS macro. Figure 2 demonstrates that the results of all paths of the mediation model were significant, after controlling for the effects of gender and age. Specifically, SES was directly and negatively associated with risk-taking behavior at a significant level (β = −0.04, *p* < 0.05), which supports H1. In terms of its indirect effect, the path from SES to psychological capital (β = 0.20, *p* < 0.001) and self-control (β = 0.08, *p* < 0.001), from psychological capital to self-control (β = 0.38, *p* < 0.001), from psychological capital to risk-taking behavior (β = −0.13, *p* < 0.001), and from self-control to risk-taking behavior (β = −0.27, *p* < 0.001) were all significant.

The results of the analysis of each mediating effect are shown in Table 2. First, the effect size of psychological capital as a mediator of the relation between SES and adolescents’ risk-taking behavior was significant [β = −0.03, CI (−0.04, −0.01)], which supports H2. Second, the mediating effect of self-control on the relation between SES and adolescents’ risk-taking behavior was significant [β = −0.02, CI (−0.03, −0.01)], which supports H3. Finally, the chain mediating effect of psychological capital and self-control on the relation between SES and adolescents’ risk-taking behavior was significant [β = −0.02, CI (−0.03, −0.01)], which supports H4. The above results indicate that psychological capital and self-control play multiple mediating roles in the relationship between SES and adolescents’ risk-taking behavior.

**TABLE 2:** The mediating effects of psychological capital and self-control.

Pathways	β	95%CI		Ratio of total effect
		Boot LLCI	Boot ULCI	
SES → Psychological capital→ Risk-taking behavior	−0.03	−0.04	−0.01	27.27%
SES→ Self-control→ Risk-taking behavior	−0.02	−0.03	−0.01	18.18%
SES→ Psychological capital→ Self-control→ Risk-taking behavior	−0.02	−0.03	−0.01	18.18%

*β refers to standardized indirect effect.*

## Discussion

### The Relationship Between Socioeconomic Status and Adolescents’ Risk-Taking Behavior

This study has used the framework of PBT to explore the relationship between socioeconomic status and adolescent risk-taking behavior and uncover its potential mechanism of action. SES was both negatively correlated with adolescents’ risk-taking behavior. Thus, the first hypothesis of this study was confirmed, meaning that in the Chinese context, SES was also negatively associated with adolescents’ risk-taking behavior, a finding consistent with results from other cultural contexts (Lee et al., 2017; Delker et al., 2018). However, it is important to note that this significant but small effect size result may indicate the need to consider additional factors associated with low SES and adolescent risk-taking behavior. Poor SES is only one external risk factor among many family related factors that reinforce adolescents’ risk-taking tendencies. The FSM explains how disadvantaged SES impacts the relationships between couples and those between parents and adolescents, parenting styles and the development of the next generation (Conger et al., 2010). The same authors point out that family economic hardship and pressure can indirectly correlate with the adaptive behavior of adolescents by influencing the behavioral and emotional functioning of parents. This is due to the fact that the economic pressure of parents indicates the parental conflicts, which directly undermines the parenting style (Conger et al., 2002). For example, harsh, inconsistent or uninvolved parenting practices can lead to emotional and behavioral problems and even cognitive impairment in the next generation, further promoting the tendency to take risks.

In addition, the inconsistencies between our findings and those of other studies may first arise from methodological differences in the calculations used to define SES, this study examines SES more comprehensively than others. Secondly, since some studies may have included closer family environmental factors, which resulting in a weakened or even insignificant effect of SES on risk-taking behavior. The presence of these inconsistencies points to the need for further research to clarify the relationship between the two. It is worth noting that even when the two mediating variables of psychological capital and self-control are introduced, the direct mediating role of SES on adolescents’ risk-taking behavior remains significant, suggesting that it has a basic role in individual psychological and behavioral development (Letourneau et al., 2013). One longitudinal study has shown that SES has a profound and lasting impact on individual development, with the SES of the main parent predictive of personal inner characteristics and problem behaviors in the next generation (Martin et al., 2010). This points to the need to explore the role of additional individual psychological characteristics in the relationship between SES and risk-taking behavior.

### The Mediating Effect of Psychological Capital

Confirming H2, the study found that psychological capital mediates SES and adolescent risk-taking behavior. Specifically, the development of individual psychological capital is related to their own SES, and a lower level of psychological capital will trigger more risk-taking behaviors. Although few studies have directly demonstrated that psychological capital mediates the relationship between SES and risk-taking among adolescents, some studies have proved that positive psychological resources played an mediating role in the relationship between the two (Layte and Whelan, 2009; Banstola et al., 2020; Liu et al., 2020). According to the reserve capacity model, low-status socioeconomic groups suffer more economic and psychological pressure, which both consumes their existing psychological resources and restricts the development of new ones, in turn increasing the occurrence of negative emotions and health risk behavior (Matthews and Gallo, 2011). It is currently known that some risk-taking behaviors (such as smoking, drinking, aggression, and unsafe sexual activity) are either socially deviant or health risk behaviors, so the theory may provide possible explanations for this mediating role. It also affords a novel perspective on the mechanism underlying the relationships between SES and the psychological and behavioral development of individuals. In addition, existing studies have demonstrated the potential for developing psychological capital. One short-term intervention in the health-risk behaviors (substance abuse and risky sex) of female participants from the lowest social strata showed that increasing levels of psychological capital significantly reduced the frequency of substance use and health-risk behaviors among this group (Rew et al., 2017). Thus, raising the psychological capital of low-SES adolescents appears an effective way of reducing risk-taking behavior.

### The Mediating Effect of Self-Control

Consistent with H3, the study confirmed self-control as another important mediator of the relationship between SES and adolescents’ risk-taking behavior. This essentially coheres with previous findings that low SES is usually associated with lower levels of self-control among adolescents, indirectly leading to an increased incidence of risk-taking behaviors that hinder physical and mental health (Brieant et al., 2020). One reason for this may be the impact of low SES on individual cognitive neurodevelopment. Studies from neurophysiology have shown that the imbalance between the long-term development of the prefrontal region of the brain and the rapid development of the limbic system in adolescence increases sensitivity to environmental stimuli at this stage (Strang et al., 2013). This disparity provides the physiological basis for risk-taking behaviors in adolescence (Mani et al., 2013). However, teenagers with deprived backgrounds may be habitually exposed to stressful and resource-poor environments, restricting the normal development of the prefrontal region and its associated function of self-control related to higher-level cognitive processing, planning, and behavioral regulation, which can easily lead to participation in high-risk behavioral activities (Casey et al., 2008). Moreover, the influence of SES on individuals’ self-control, physical aggression, anti-social and other maladaptive behaviors has an intergenerational transmission effect. In addition to genetic factors, adults with low SES may lack the knowledge and experience that underpin parenting skills, leading to lower self-control and increased risk-taking behavior in their adolescent offspring (Boutwell and Beaver, 2010; Gao, 2014). Indeed, previous studies have proven that parenting style is closely related to adolescents’ self-control, adopting positive parenting style is conducive to the development of adolescent self-control (Li J. B. et al., 2019), and improving individual self-control can effectively reduce the occurrence of risk-taking behavior (Xu, 2005). It also reminds us that developing self-control can protect adolescents from the negative psychological and behavioral effects of low SES, including engagement in risky behaviors.

### The Chain Mediating Effect of Psychological Capital and Self-Control

Consistent with our hypothesis, the study found that psychological capital and self-control mediated the relationship between SES and adolescents’ risk-taking behavior is not only independent but sequential. The sequential effect indicated that psychological capital also mediates the link between SES and self-control. These findings suggest that SES reflects the ability of families to provide material and psychological resources for children (Masarik and Conger, 2017), and also demonstrate the relationship between psychological capital and self-control. In other words, the lower the SES, the lower the level of psychological resources such as self-efficacy, optimism and hope, which means that fewer resources are available for self-control, and the possibility of developing new resources is also reduced. As a result, the individual is less able to restrain their impulses and displays a higher tendency toward risk-taking behavior (Gottfredson and Hirschi, 1990). Given the relative difficulty of changing SES, this discovery points to interventions focused on developing psychological capital as a potential means of enhancing self-control and lowering the prevalence of risk-taking. On one hand, it is necessary to raise parents’ awareness of their role in improving their parenting skills and educating their offspring to enhance their cognitive development. On the other, schools should improve psychological capital and self-control *via* measures such as group counseling, behavior training, and so on (He and Shi, 2015). This may well help reduce adolescents’ negative risk-taking tendencies and promote their healthy development.

### Limitations

Although this study has provided a detailed investigation of the factors involved in risk-taking behavior during adolescence, it contains several limitations. First of all, as a cross-sectional study, it was unable to capture any changes that may occur in such behavior over time. However, an interactive model of SES, family processes, and individual development has been proposed, emphasizing the cumulative effect of the negative consequences of low SES (Conger et al., 2010). Thus, the influence of SES on individual risk-taking behavior may be explored in a long-term follow-up study to better clarify the relationship between the two. Secondly, in addition to SES, there may be many more closely related environmental factors associated with adolescents’ risk-taking behavior have not been considered in this study. The significant but weak effect of SES on risk-taking behavior suggested that additional factors are implicated in the relationship. However, this study has only considered the link between SES and negative risk-taking behavior in terms of the healthy development of adolescents, the mechanism underlying positive risk-taking behavior in adolescents is still unclear, which needs more discussion in the future. Finally, this study’s use of self-report questionnaires required the participants to disclose sensitive information. Although participants were anonymized, the pressure to provide socially desirable responses may have impacted the accuracy of the data. This points to the need to observe authentic behavior *via* experimental methods in order to evaluate the risk-taking tendencies of adolescents.

## Conclusion

In conclusion, this study has demonstrated that psychological capital and self-control are potential mediating variables in the relationship between SES and adolescents’ risk-taking behavior. In particular, psychological capital and self-control can mediate this relationship both independently and sequentially. The current study has thus contributed to the expansion of theoretical research into the mechanism by which such behavior is mediated, and explored further factors involved. Adolescents who lack material and psychological resources will typically display decreased self-control and increased levels of negative risk-taking. The conclusion provides an intervention approach for adolescents to prevent and reduce risk-taking behavior in the future by improving the supply of external and internal resources.

## Data Availability Statement

All the raw data for this study can be found here: https://doi.org/10.6084/m9.figshare.15134391.v1.

## Ethics Statement

The studies involving human participants were reviewed and approved by the Ethics Committee of Normal College of Shihezi University. Written informed consent to participate in this study was provided by the participants’ legal guardian/next of kin.

## Author Contributions

HM, YZ, and XJ collected the data. XJ analyzed the data and drafted the manuscript. HZ and GS revised the manuscript. All authors have read and approved the final submitted version of the manuscript.

## Conflict of Interest

The authors declare that the research was conducted in the absence of any commercial or financial relationships that could be construed as a potential conflict of interest.

## Publisher’s Note

All claims expressed in this article are solely those of the authors and do not necessarily represent those of their affiliated organizations, or those of the publisher, the editors and the reviewers. Any product that may be evaluated in this article, or claim that may be made by its manufacturer, is not guaranteed or endorsed by the publisher.

## References

[B1] Adler N. E. Epel E. S. Castellazzo G. Ickovics J. R. (2000). Relationship of subjective and objective social status with psychological and physiological functioning: preliminary data in healthy white women. *Health Psychol.* 19 586–592. 10.1037/0278-6133.19.6.586 11129362

[B2] Banstola R. S. Ogino T. Inoue S. (2020). Self-esteem, perceived social support, social capital, and risk-behavior among urban high school adolescents in Nepal. *SSM Popul. Health* 11:100570. 10.1016/j.ssmph.2020.100570 32258358 PMC7115101

[B3] Baumeister R. F. Vohs K. D. Tice D. M. (2007). The strength model of self-control. *Curr. Dir. Psychol. Sci.* 16 351–355. 10.1111/j.1467-8721.2007.00534.x

[B4] Ben-Zur H. Zeidner M. (2009). Threat to life and risk-taking behaviors: a review of empirical findings and explanatory models. *Pers. Soc. Psychol. Rev.* 13 109–128. 10.1177/1088868308330104 19193927

[B5] Bersamin M. Paschall M. J. Fisher D. A. (2017). School-based health centers and adolescent substance use: moderating effects of race/ethnicity and socioeconomic status. *J. Sch. Health* 87 850–857. 10.1111/josh.12559 29023835 PMC5654608

[B6] Boutwell B. B. Beaver K. M. (2010). The intergenerational transmission of low self-control. *J. Res. Crime Delinq.* 47 174–209. 10.1177/0022427809357715

[B7] Bradley R. H. Corwyn R. F. (2002). Socioeconomic status and child development. *Annu. Rev. Psychol.* 21 371–399. 10.1146/annurev.psych.53.100901.135233 11752490

[B8] Brieant A. Peviani K. M. Lee J. E. King-Casas B. Kim-Spoon J. (2020). Socioeconomic risk for adolescent cognitive control and emerging risk-taking behaviors. *J. Res. Adolesc.* 31 71–84. 10.1111/jora.12583 32951287 PMC8162917

[B9] Casey B. Jones R. M. Hare T. A. (2008). The adolescent brain. *Ann. N. Y. Acad. Sci..* 1124 111–126. 10.1196/annals.1440.010 18400927 PMC2475802

[B10] Chen Y. H. Cheng G. Guan Y. S. Zhang D. J. (2014). The mediating effects of subjective social status on the relations between self-esteem and socioeconomic status for college students. *Psychol. Dev. Educ.* 30 594–600. 10.16187/j.cnki.issn1001-4918.2014.06.005

[B11] Cheung N. W. T. (2014). Low self-control and co-occurrence of gambling with substance use and delinquency among Chinese adolescents. *J. Gambl. Stud.* 30 105–124. 10.1007/s10899-012-9351-8 23224660

[B12] Conger R. D. Conger K. J. Martin M. J. (2010). Socioeconomic status, family processes, and individual development. *J. Marriage Fam.* 72 685–704. 10.1111/j.1741-3737.2010.00725.x 20676350 PMC2910915

[B13] Conger R. D. Elder G. H. (1996). *Families in Troubled Times: Adapting to Change in Rural America.* Hawthorne, NY: Aldine de Gruyter, 10.2307/2580396

[B14] Conger R. D. Wallace L. E. Sun Y. Simons R. L. Mcloyd V. C. Brody G. H. (2002). Economic pressure in African american families: a replication and extension of the family stress model. *Dev. Psychol.* 38 179–193. 10.1037/0012-1649.38.2.179 11881755

[B15] Cui Y. Q. Lou C. H. (2019). A review of the association between adolescent social capital and health risk behaviors. *Chin. J. Health Educ.* 35 1117–1121. 10.16168/j.cnki.issn.1002-9982.2019.12.015

[B16] Delker B. C. Bernstein R. E. Laurent H. K. (2018). Out of harm’s way: secure versus insecure-disorganized attachment predicts less adolescent risk taking related to childhood poverty. *Dev. Psychopathol.* 30 283–296. 10.1017/S0954579417000621 28508736

[B17] Duell N. Steinberg L. (2020). Differential correlates of positive and negative risk taking in adolescence. *J. Youth Adolesc.* 49 1162–1178. 10.1007/s10964-020-01237-7 32335842 PMC7242164

[B18] Farley J. P. Kim-Spoon J. (2017). Parenting and adolescent self-regulation mediate between family socioeconomic status and adolescent adjustment. *J. Early Adolesc.* 37 502–524. 10.1177/0272431615611253 28348448 PMC5365151

[B19] Finch J. Farrell L. J. Waters A. M. (2020). Searching for the HERO in youth: does psychological capital (PsyCap) predict mental health symptoms and subjective wellbeing in Australian school-aged children and adolescents? *Child Psychiatry Hum. Dev.* 51 1025–1036. 10.1007/s10578-020-01023-3 32666426 PMC7358995

[B20] Gao W. B. (2014). “A cross-sectional study on self-control: growth curve and intergenerational transmission. Stress and Mental Health [Conference presentation],” in *The 4th Annual Mental Health Academic Conference*, Jiangsu.

[B21] Gardner M. Steinberg L. (2005). Peer influence on risk taking, risk preference, and risky decision making in adolescence and adulthood: an experimental study. *Dev. Psychol.* 41 625–635. 10.1037/0012-1649.41.4.625 16060809

[B22] Goosby J. B. (2007). Poverty duration, maternal psychological resources, and adolescent socioemotional outcomes. *J. Fam. Issues* 28 1113–1134. 10.1177/0192513X07300712

[B23] Gottfredson M. R. Hirschi T. (1990). *A General Theory of Crime.* Stanford, CA: Stanford University Press, 10.2307/1964276

[B24] Gullone E. Moore S. Moss S. Boyd C. (2000). The adolescent risk-taking questionnaire: development and psychometric evaluation. *J. Adolesc. Res.* 15 231–250. 10.1177/0743558400152003

[B25] He L. Shi Z. B. (2015). Intervention studies based on the strength model of self-control: a review. *Chin. J. Ment. Health* 29 366–371. 10.3969/j.issn.1000-6729.2015.05.012

[B26] Hong R. Y. Paunonen S. V. (2009). Personality traits and health-risk behaviours in university students. *Eur. J. Pers.* 23 675–696. 10.1002/per.736

[B27] Jessor R. (1987). Problem-behavior theory, psychosocial development, and adolescent problem drinking. *Br. J. Addict.* 82 331–342. 10.1111/j.1360-0443.1987.tb01490.x 3472582

[B28] Johnson A. H. (2012). Resilience builder program for children and adolescents: enhancing social competence and self-regulation, a cognitive−behavioral group approach by Alvord, M. K., Sucker, B., and Grados, J. J. *Social Work Groups* 35 393–395. 10.1080/01609513.2012.656530

[B29] Kraus M. W. Horberg E. J. Goetz J. L. Keltner D. (2011a). Social class rank, threat vigilance, and hostile reactivity. *Pers. Soc. Psychol. Bull.* 37 1376–1388. 10.1177/0146167211410987 21653579

[B30] Kraus M. W. Piff P. K. Keltner D. (2011b). Social class as culture the convergence of resources and rank in the social realm. *Cur. Dir. Psychol. Sci.* 20 246–250. 10.1177/0963721411414654

[B31] Lansford J. E. Malone P. S. Stevens K. I. Dodge K. A. Bates J. E. Pettit G. S. (2006). Developmental trajectories of externalizing and internalizing behaviors: factors underlying resilience in physically abused children. *Dev. Psychopathol.* 18 35–55. 10.1017/S0954579406060032 16478551 PMC2772062

[B32] Layte R. Whelan C. L. (2009). Explaining social class inequalities in smoking: the role of education, self-efficacy, and deprivation. *Eur. Sociol. Rev.* 25 399–410. 10.1093/esr/jcn022

[B33] Lee H. J. Park S. Kim C. I. Choi D. W. Lee J. S. Oh S. M. (2013). The association between disturbed eating behavior and socioeconomic status: the online Korean adolescent panel survey (on APS). *PLoS One* 8:e0057880. 10.1371/journal.pone.0057880 23472117 PMC3589486

[B34] Lee J. O. Cho J. Yoon Y. Bello M. S. Leventhal A. M. (2017). Developmental pathways from parental socioeconomic status to adolescent substance use: alternative and complementary reinforcement. *J. Youth Adolesc.* 47 334–348. 10.1007/s10964-017-0790-5 29188410 PMC5790622

[B35] Letourneau N. L. Duffett-Leger L. Levac L. Watson B. Young-morris C. (2013). Socioeconomic status and child development: a meta-analysis. *J. Emot. Behav. Disord.* 21 211–224. 10.1177/1063426611421007

[B36] Li J. B. Willems Y. E. Stok F. M. Deković M. Bartels M. Finkenauer C. (2019). Parenting and self-control across early to late adolescence: a three-level meta-analysis. *Perspect. Psychol. Sci.* 14 967–1005. 10.1177/1745691619863046 31491364

[B37] Li X. X. Ren Z. H. Hu X. Y. Guo Y. Y. (2019). Why are undergraduates from lower-class families more likely to experience social anxiety? ——the multiple mediating effects of psychosocial resources and rejection sensitivity. *Psychol. Sci.* 42 1354–1360. 10.16719/j.cnki.1671-6981.20190611

[B38] Liu G. Z. Zhang D. J. Zhu Z. G. Li J. J. Chen X. (2020). The effect of family socioeconomic status on adolescents’ problem behaviors:the chain mediating role of parental emotional warmth and belief in a just world. *Psychol. Dev. Educ.* 36 240–248. 10.16187/j.cnki.issn1001-4918.2020.02.13

[B39] Liu L. Wang N. Tian L. (2019). The parent-adolescent relationship and risk-taking behaviors among Chinese adolescents: the moderating role of self-control. *Front. Psychol.* 10:542. 10.3389/fpsyg.2019.00542 30949091 PMC6435964

[B40] Lu X. Y. (2002). *A Report on Social Classes in Contemporary China.* Beijing: Social Sciences Academic Press.

[B41] Ludwig K. B. Pittman J. F. (1999). Adolescent prosocial values and self-efficacy in relation to delinquency, risky sexual behavior, and drug use. *Youth Soc.* 30 461–482. 10.1177/0044118X99030004004

[B42] Luthans F. Youssef C. Avolio B. (2007). *Psychological Capital: Developing the Human Competitive Edge.* New York, NY: Oxford University Press, 107–108. 10.1093/acprof:oso/9780195187526.001.0001

[B43] Luthans F. Youssef C. M. Avolio B. J. (2015). *Psychological Capital and Beyond.* New York, NY: Oxford University Press.

[B44] Mabekoje S. O. (2010). *Emotional Intelligence and Self-Regulation Among School-Going Adolescents: Self-Efficacy as a Mediator.* Nigeria: Olabisi Onabanjo University.

[B45] Malti T. Noam G. G. (2009). A developmental approach to the prevention of adolescent’s aggressive behavior and the promotion of resilience. *Int. J. Dev. Sci.* 3 235–246. 10.3233/DEV-2009-3303

[B46] Mani A. Mullainathan S. Shafir E. Zhao J. (2013). Poverty impedes cognitive function. *Science* 341 976–980. 10.1126/science.1238041 23990553

[B47] Margot P. Tineke O. Wilma V. (2017). Behavioral control and reward sensitivity in adolescents’ risk taking behavior: a longitudinal trails study. *Front. Psychol.* 8:231. 10.3389/fpsyg.2017.00231 28261148 PMC5313488

[B48] Martin M. J. Conger R. D. Schofield T. J. Dogan S. J. Widaman K. F. Donnellan M. B. (2010). Evaluation of the interactionist model of socioeconomic status and problem behavior: a developmental cascade across generations. *Dev. Psychopathol.* 22 695–713. 10.1017/S0954579410000374 20576188 PMC2892802

[B49] Masarik A. S. Conger R. D. (2017). Stress and child development: a review of the family stress model. *Curr. Opin. Psychol.* 13 85–90. 10.1016/j.copsyc.2016.05.008 28813301

[B50] Matthews K. A. Gallo L. C. (2011). Psychological perspectives on pathways linking socioeconomic status and physical health. *Annu. Rev. Psychol.* 62 501–530. 10.1146/annurev.psych.031809.130711 20636127 PMC3121154

[B51] Mcghee R. L. Ehrler D. J. Buckhalt J. A. Phillips C. (2012). The relation between five-factor personality traits and risk-taking behavior in preadolescents. *Psychol* 3 558–561. 10.4236/psych.2012.38083

[B52] Mueller C. W. Parcel T. L. (1981). Measures of socioeconomic status: alternatives and recommendations. *Child Dev.* 52 13–30. 10.1111/1467-8624.ep8861601

[B53] Özmen O. Sümer Z. H. (2011). Predictors of risk-taking behaviors among Turkish adolescents. *Pers. Individ. Diff.* 50 4–9. 10.1016/j.paid.2010.07.015

[B54] Piotrowska P. J. Stride C. B. Croft S. E. Rowe R. (2015). Socioeconomic status and antisocial behaviour among children and adolescents: a systematic review and meta-analysis. *Clin. Psychol. Rev.* 35 47–55. 10.1016/j.cpr.2014.11.003 25483561

[B55] Ren X. C. Lv Q. Z. (2016). The present situation, cause and resolution of juvenile delinquency. *Chin. Youth Stud.* 244 103–108. 10.19633/j.cnki.11-2579/d.2016.06.016

[B56] Rew L. Powell T. Brown A. Becker H. Slesnick N. (2017). An intervention to enhance psychological capital and health outcomes in homeless female youths. *West J. Nurs. Res.* 39 356–373. 10.1177/0193945916658861 27411974

[B57] Rolison M. R. Scherman A. (2003). College student risk-taking from three perspectives. *Adolescence* 38 689–704. 15053495

[B58] Rosenthal D. Moore S. Flynn I. (2010). Adolescent self-efficacy, self-esteem and sexual risk-taking. *J. Commun. Appl. Soc. Psychol.* 1 77–88. 10.1002/casp.2450010203

[B59] Schelleman-Offermans K. Massar K. (2020). Explaining socioeconomic inequalities in self-reported health outcomes: the mediating role of perceived life stress, financial self-reliance, psychological capital, and time perspective orientations. *PLoS One* 15:e0243730. 10.1371/journal.pone.0243730 33370306 PMC7769277

[B60] Shang S. J. Bai B. Y. Zhong N. (2016). Family social class and meaning in life: mediating of basic psychological need satisfaction. *Chin. J. Clin. Psychol.* 24 1108–1111. 10.16128/j.cnki.1005-3611.2016.06.032

[B61] Shoenberger N. Rocheleau G. C. (2017). Effective parenting and self-control: difference by gender. *Women Crim. Justice* 27 271–286. 10.1080/08974454.2016.1261071

[B62] Smith A. R. Chein J. Steinberg L. (2014). Peers increase adolescent risk taking even when the probabilities of negative outcomes are known. *Dev. Psychol.* 50 1564–1568. 10.1037/a0035696 24447118 PMC4305434

[B63] Steinberg L. (2008). A social neuroscience perspective on adolescent risk-taking. *Dev Rev.* 28 78–106. 10.1016/j.dr.2007.08.002 18509515 PMC2396566

[B64] Steinberg L. (2010). Risk taking in adolescence: what changes, and why? *Ann. N. Y. Acad. Sci.* 1021 51–58. 10.1196/annals.1308.005 15251873

[B65] Steinberg L. (2016). *The Adolescent Brain. Adolescence.* Philadelphia: Temple University, 51–64.

[B66] Steinberg L. Icenogle G. Shulman E. P. Breiner K. Chein J. Bacchini D. (2017). Around the world, adolescence is a time of heightened sensation seeking and immature self-regulation. *Dev. Sci.* 21:e12532. 10.1111/desc.12532 28150391 PMC6473801

[B67] Strang N. M. Chein J. M. Laurence S. (2013). The value of the dual systems model of adolescent risk-taking. *Front. Hum. Neurosci.* 7:223. 10.3389/fnhum.2013.00223 23750132 PMC3664310

[B68] Tan J. J. X. Kraus M. W. (2015). Lay theories about social class buffer lower-class individuals against poor self-rated health and negative affect. *Pers. Soc. Psychol. Bull.* 41 446–461. 10.1177/0146167215569705 25634909

[B69] Tan S. H. Guo Y. Y. (2008a). A limited resource model of self-control and the relevant studies. *Chin. J. Clin. Psychol.* 16 309–311. 10.3969/j.issn.1005-3611.2008.03.027

[B70] Tan S. H. Guo Y. Y. (2008b). Revision of self-control scale for chinese college students. *Chin. J. Clin. Psychol.* 16 468–470. 10.16128/j.cnki.1005-3611.2008.05.022

[B71] Tangney J. P. Baumeister R. T. Boone A. L. (2004). High self-control predicts good adjustment, less pathology, better grades, and interpersonal success. *J. Pers.* 72 271–324. 10.1111/j.0022-3506.2004.00263.x 15016066

[B72] Tian L. M. Liu L. L. Yuan J. C. Shan N. Wu Y. L. (2018). Effects of family functioning on adolescent risk-taking behavior: the sequential mediating effects of self-control ability and deviant peer affiliation. *Psychol. Dev. Educ.* 34 361–368. 10.16187/j.cnki.issn1001-4918.2018.03.13

[B73] Tice D. M. Baumeister R. F. Shmueli D. Muraven M. (2007). Restoring the self: positive affect helps improve self-regulation following ego depletion. *J. Exp. Soc. Psychol.* 43 379–384. 10.1016/j.jesp.2006.05.007

[B74] Titterton M. Smart H. (2010). Risk, resilience and vulnerability in children and adolescents in relation to long-term conditions: the example of eastern Europe and central Asia. *J. Nurs. Healthc. Chronic Illn.* 2 153–163. 10.1111/j.1752-9824.2010.01055.x

[B75] Vukovic D. S. Bjegovic V. M. (2007). Brief report: risky sexual behavior of adolescents in belgrade: association with socioeconomic status and family structure. *J. Adolesc.* 30 869–877. 10.1016/j.adolescence.2007.06.005 17673284

[B76] Wang B. Tian L. M. Dong X. Y. (2018). The relationship between family functioning and adolescent negative risk-taking behavior: a moderated mediating model. *Psychol. Dev. Educ.* 34 146–154. 10.16187/j.cnki.issn1001-4918.2018.02.03

[B77] Xiong J. M. Hai M. Huang F. Xin L. Xu Y. (2020). Family cumulative risk and mental health in chinese adolescents: the compensatory and moderating effects of psychological capital. *Psychol. Dev. Educ.* 36 94–102. 10.16187/j.cnki.issn1001-4918.2020.01.11

[B78] Xu H. T. (2005). A research summary on relationship between low self-control and deviant acts. *Chin. J. Spec. Educ.* 11 61–64. 10.3969/j.issn.1007-3728.2005.05.013

[B79] Yuan Z. K. Wen X. T. (2019). Study design and prevalence of health risk behaviors among Chinese adolescents. *Chin. J. Sch. Health* 40 638–640. 10.16835/j.cnki.1000-9817.2019.04.046

[B80] Zhang C. Zhang L. J. Shang L. (2011). Reliability and validity of adolescent risk-taking questionnaire-risk behavior scale (arq-rb) in middle school students. *Chin. J. Ment. Health* 25 636–640.

[B81] Zhang K. Zhang S. Dong Y. H. (2010). Positive psychological capital: measurement and relationship with mental health. *Stud. Psychol. Behav.* 8 58–64.

[B82] Zheng H. L. Luo D. H. (2009). The role of low self-control and family socioeconomic status in juvenile delinquent——an empirical study on the causes of juvenile delinquent in China. *Youth Stud.* 366 10–17, 94.

[B83] Zhou C. Y. Guo Y. Y. (2013). Impact of family social status on mental health:mediating role of belief in a just world. *Chin. J. Clin. Psychol.* 21 636–640. 10.16128/j.cnki.1005-3611.2013.04.017

